# Supplementary material for: Elimination of SOX2/OCT4-Associated Prostate Cancer Stem Cells Blocks Tumor Development and Enhances Therapeutic Response
Source: Cancers (Basel). 2019 Sep 8;11(9):1331. doi: 10.3390/cancers11091331 (PMC6769476; doi:10.3390/cancers11091331)
Supplement: Supplementary file 1 [file cancers-11-01331-s001.pdf]

cancers

MDPI

Supplementary Materials

Elimination of SOX2/OCT4-Associated Prostate Cancer Stem Cells Blocks Tumor Development and Enhances Therapeutic Response

Prasanna Kumar Vaddi, Mark A. Stamnes, Huojun Cao and Songhai Chen

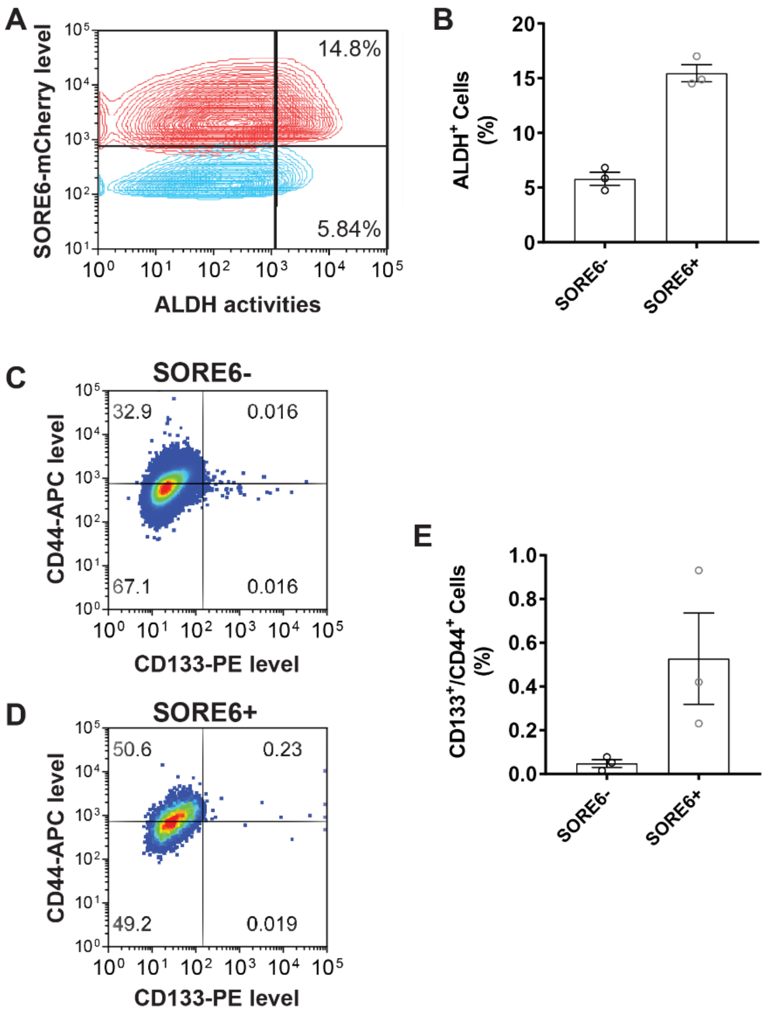

Figure S1. Comparison of CSC populations identified by the SORE6 reporter, ALDH and CD44/CD133. (**A**) A representative flow cytometry analysis of PC3 cells expressing SORE6-mCherry for ALDH activities. Top panel, SORE6-mCherry^{+}; bottom panel, SORE6-mCherry^{-}. The percentage of cells expressing high level of ALDH activities are indicated. (**B**) Quantitative data from three independent experiments showing percentage of SORE6^{-} and SORE6^{+} cells expressing ALDH. in a panel of PCa cells identified by flow cytometry. (**C–E**) Flow cytometry analysis of CD44 and CD133 expression in SORE6^{-} and SORE6^{+} PC3 cells. Representative flow cytometry plots are shown in C (SORE6^{-}) and D (SORE6^{+}). Quantitative data from three independent experiments showing percentage of SORE6^{-} and SORE6^{+} cells expressing CD44^{+}/CD133^{+} are shown in E.

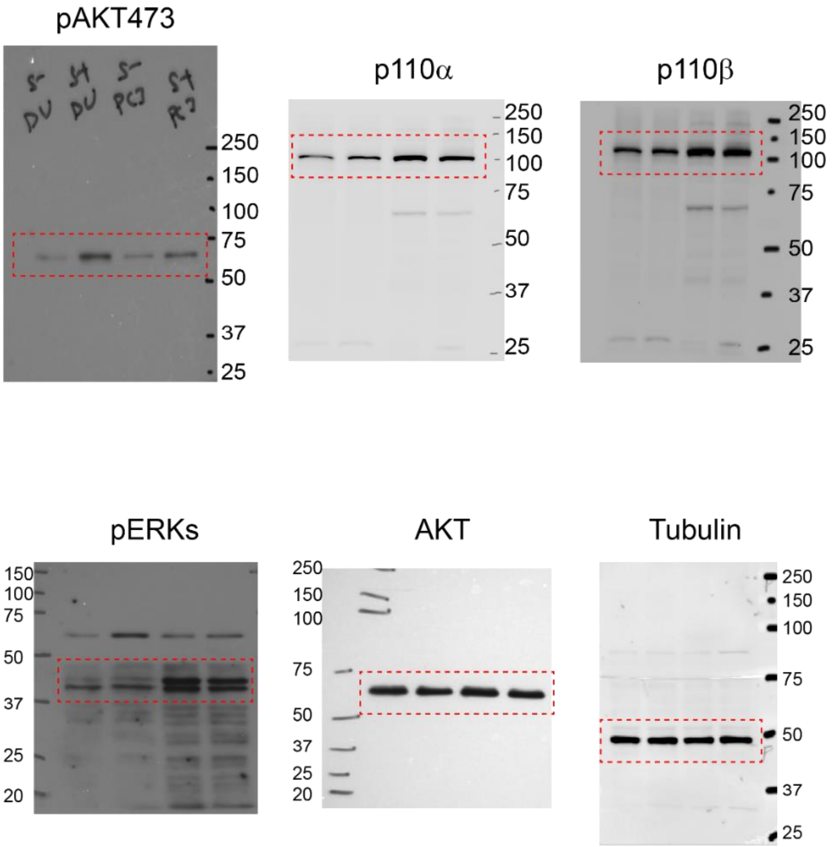

Figure S2: The original blots for Figure 5A. The cropped bands were indicated by boxes.

4 dx3454e4e43343ew1 fdas

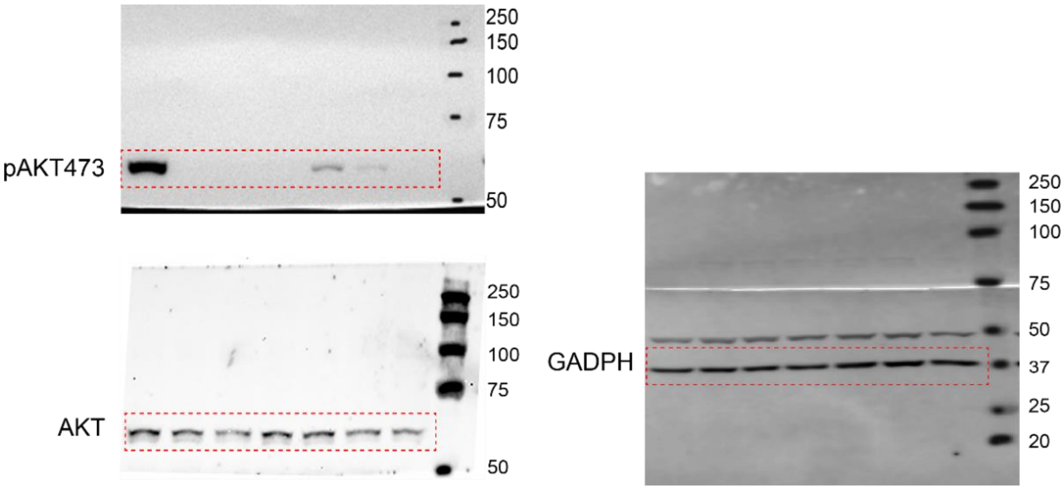

Figure S3: The original blots for Figure 5B. The cropped bands were indicated by boxes.

Table S1. Altered gene expression in SORE6$^+$ cells compared to SORE6$^-$ cells.

Gene ID	Average Log$_2$ Change	*p* Value
LINC00261	2.3000	3.47E-08
HSPB8	2.2710	0.0000012
SCNN1B	1.5185	0.000143703
KRT37	1.4521	0.000000962
DYNC1I1	1.2261	0.000683332
IGF2	1.1711	0.00095664
TUBB4A	1.1432	0.000622161
B4GALNT1	1.1341	0.000485262
PANX2	1.0928	6.07E-18
GALNT4	1.0173	0.0000211
PPFIA4	0.9922	0.000032
IFI27	0.9876	0.0000508
ALDOC	0.9673	0.000407027
FAM83A	0.9551	1.68E-10
LOC154761	0.9377	7.17E-08
SERPINE1	0.9348	0.000383171
KLK5	0.9266	0.000292768
EFR3B	0.9105	6.21E-10
CEACAM5	0.9006	0.00049199
MLLT11	0.8964	8.04E-13
CASP14	0.8757	5.98E-16
PLAT	0.8675	0.0000228
MAP1B	0.8644	9.57E-37
CD68	0.8594	0.00000293
VIM	0.8568	1.05E-08
SOD3	0.8356	1.91E-22
NDRG1	0.8355	5.09E-11
FLNC	0.8324	2.16E-51
RIMKLA	0.8273	1.12E-25
STC1	0.8180	2.01E-10
KCNAB2	0.8001	0.0000313
CEACAM6	0.7801	5.76E-08
HEPHL1	0.7795	0.000526229
BSCL2	0.7725	0.0000213
BNIP3	0.7702	0.0000838
MSMP	0.7594	2.61E-19
IL6	−0.8332	1.38E-16
CYP1B1	−0.8927	5.4E-18
TSPAN2	−0.9127	0.000798727
VCAN	−0.9506	1.49E-15
HIST2H2BF	−0.9728	0.0000448
SYT12	−0.9767	0.000636366
TRIM31	−0.9972	0.0000933
FABP4	−1.0309	0.0000275
IGFBP5	−1.0993	6.38E-17
DHRS2	−1.2561	0.00000151
CYP1A1	−1.2565	0.000928116
TC2N	−1.3446	0.000447963
HIST2H4B	−1.3807	0.00000391
LOC105370526	−1.5210	0.000162024
KCNK5	−1.6045	0.000814113
MGP	−1.9173	7.19E-09
SMIM11A	−2.4297	6.51E-11
MS4A7	−2.4700	2.01E-11
ARC	−3.0136	3.4E-24
RRAD	−3.4031	0.00000121

© 2019 by the authors. Licensee MDPI, Basel, Switzerland. This article is an open access article distributed under the terms and conditions of the Creative Commons Attribution (CC BY) license (http://creativecommons.org/licenses/by/4.0/).
